# Unmasking a Rare Venous-Predominant Arteriovenous Malformation: A Case of Recurrent Subarachnoid Hemorrhage Successfully Treated With Stereotactic Radiosurgery

**DOI:** 10.7759/cureus.80265

**Published:** 2025-03-08

**Authors:** Moustafa A Mansour, Hamdi N Mostafa

**Affiliations:** 1 Neurosurgery, Nasser Institute for Research and Treatment, Cairo, EGY

**Keywords:** developmental venous anomaly, stereotactic radiosurgery, subarachnoid hemorrhage, vascular neurosurgery, venous-predominant arteriovenous malformation

## Abstract

Arteriovenous malformations (AVMs) are a leading cause of intracranial hemorrhage in young adults, yet venous-predominant AVMs remain exceedingly rare and diagnostically challenging, often mimicking benign developmental venous anomalies (DVAs). We present the case of an 18-year-old male who suffered from recurrent subarachnoid hemorrhages due to a venous-predominant AVM initially misidentified as a DVA. Given the lesion’s deep location and high surgical risk, stereotactic radiosurgery (SRS) was selected as the primary treatment modality. Over a 64-month follow-up, the patient demonstrated progressive obliteration of the AVM, culminating in complete resolution on conventional angiography at 55 months post-SRS. This case underscores the critical role of advanced imaging in distinguishing rare AVM subtypes, the importance of multidisciplinary decision-making, and the long-term efficacy of SRS in managing high-risk vascular lesions.

## Introduction

Arteriovenous malformations (AVMs) are congenital vascular anomalies characterized by abnormal connections between arteries and veins, bypassing the capillary bed. These lesions are a significant cause of intracranial hemorrhage, particularly in young adults, and can lead to severe neurological deficits or even death if not managed appropriately [1,2]. While AVMs are typically arterial-predominant, venous-predominant AVMs are exceedingly rare and can mimic other vascular anomalies, such as developmental venous anomalies (DVAs), making their diagnosis and management particularly challenging [3].

Subarachnoid hemorrhage (SAH) is a common presentation of AVMs, often heralded by a sudden, severe thunderclap headache, as seen in this case. The management of AVMs, especially those in deep or eloquent brain regions, requires a multidisciplinary approach, balancing the risks of intervention against the natural history of the disease [4]. Stereotactic radiosurgery (SRS) has emerged as a viable treatment option for high-risk AVMs, offering a non-invasive alternative to surgical resection with favorable long-term outcomes [5].

Here, we present a case of an 18-year-old male with a rare venous-predominant AVM mimicking a DVA, who experienced recurrent SAH and was successfully treated with SRS. This case highlights the diagnostic challenges, the importance of multidisciplinary decision-making, and the long-term efficacy of SRS in managing complex AVMs.

## Case presentation

An 18-year-old male presented to the emergency department with a sudden, severe thunderclap headache, described as the worst headache of his life. The pain was diffuse but predominantly localized to the right frontal region, accompanied by nausea and vomiting. He had no prior history of similar headaches, trauma, or known neurological conditions.

**Figure 1:**
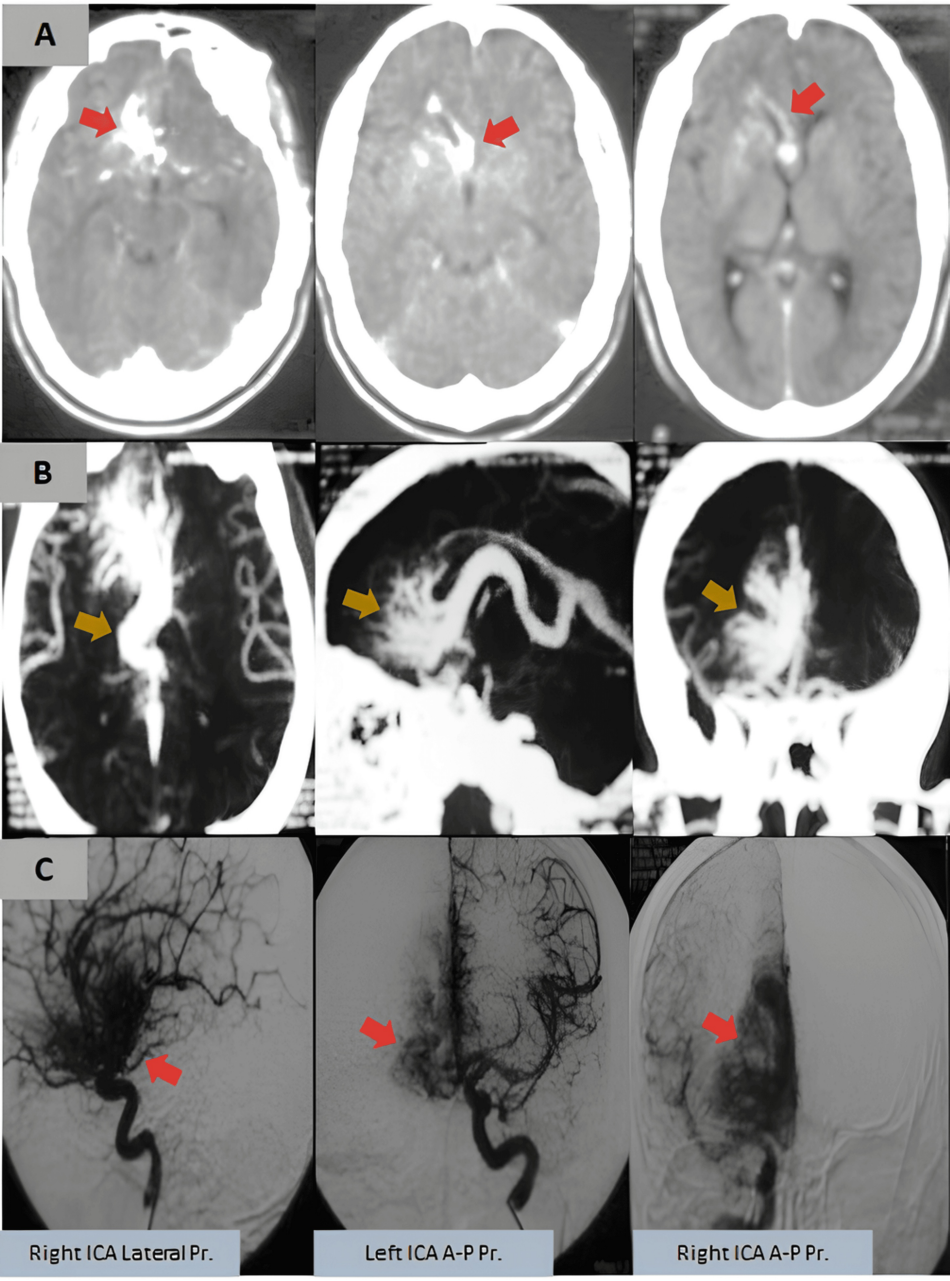
Imaging at initial presentation (A) Non-contrast CT brain images at initial presentation, revealing a subarachnoid hemorrhage in the right frontal region without intraventricular extension (arrows). (B) CT angiography and (C) digital subtraction angiography demonstrating a rare venous-predominant arteriovenous malformation mimicking an atypical developmental venous anomaly (arrows). ICA, internal carotid artery

On arrival, he was alert and oriented, with stable vital signs. However, a non-contrast CT scan of the brain revealed evidence of SAH in the right frontal region (Figure 1A). A CT angiogram (CTA) and digital subtraction angiography (DSA) were performed, which demonstrated a rare subtype of AVM with a venous-predominant nidus, mimicking an atypical arteriovenous DVA (Figures 1B, 1C). Given the hemorrhagic presentation, the patient was admitted to the neurology service for close monitoring and further evaluation.

Over the next few weeks, he made a complete recovery with conservative management, including supportive care and blood pressure control. The patient refused to discuss any kind of intervention at this time. However, one month after discharge, he suffered a second hemorrhagic episode, presenting again with a severe headache and vomiting. Repeat imaging confirmed recurrent SAH, prompting a multidisciplinary discussion between neurosurgery and radiation oncology. Due to the deep location and diffuse nature of the AVM, surgical resection was deemed high risk based on the Spetzler-Martin grading system (4/5 points) and Lawton supplementary scale (2/5 points) for cerebral AVMs besides the Toronto scale (31.6% probability of neurologic deficit with surgery) for the assessment of surgery-related risks for cerebral AVMs. Therefore, SRS was selected as the primary treatment modality.

The patient underwent linac-based SRS targeting the right frontal venous-predominant AVM. The treatment plan included a target volume of 12.5 cc, a marginal dose of 25.0 Gy, and an isodose level of 80%, with a maximum optic chiasm dose of 15.8 Gy. Following the procedure, he was discharged home with instructions for close outpatient follow-up.

At his three-month follow-up, the patient reported moderate headache and intermittent vomiting, prompting initiation of steroids and diuretics. By five months post-SRS, his symptoms were improving, allowing gradual tapering of medications. However, at seven months, he experienced a recurrence of severe headache and vomiting, necessitating a brief course of steroids before achieving symptom resolution by the eighth month.

**Figure 2:**
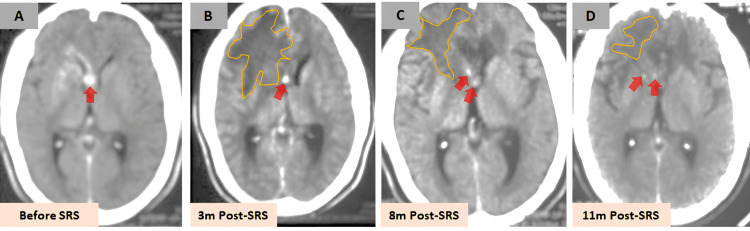
Serial CT follow-up post-SRS Serial CT images before and after SRS demonstrating a progressive treatment response. (A) Baseline CT before SRS. (B) At three months post-SRS, there is a slight reduction in arteriovenous malformation nidus size (red arrow), with significant vasogenic edema in the right frontal region (yellow outline). (C, D) Both the edema and nidus gradually decreased over time. SRS, stereotactic radiosurgery

**Figure 3:**
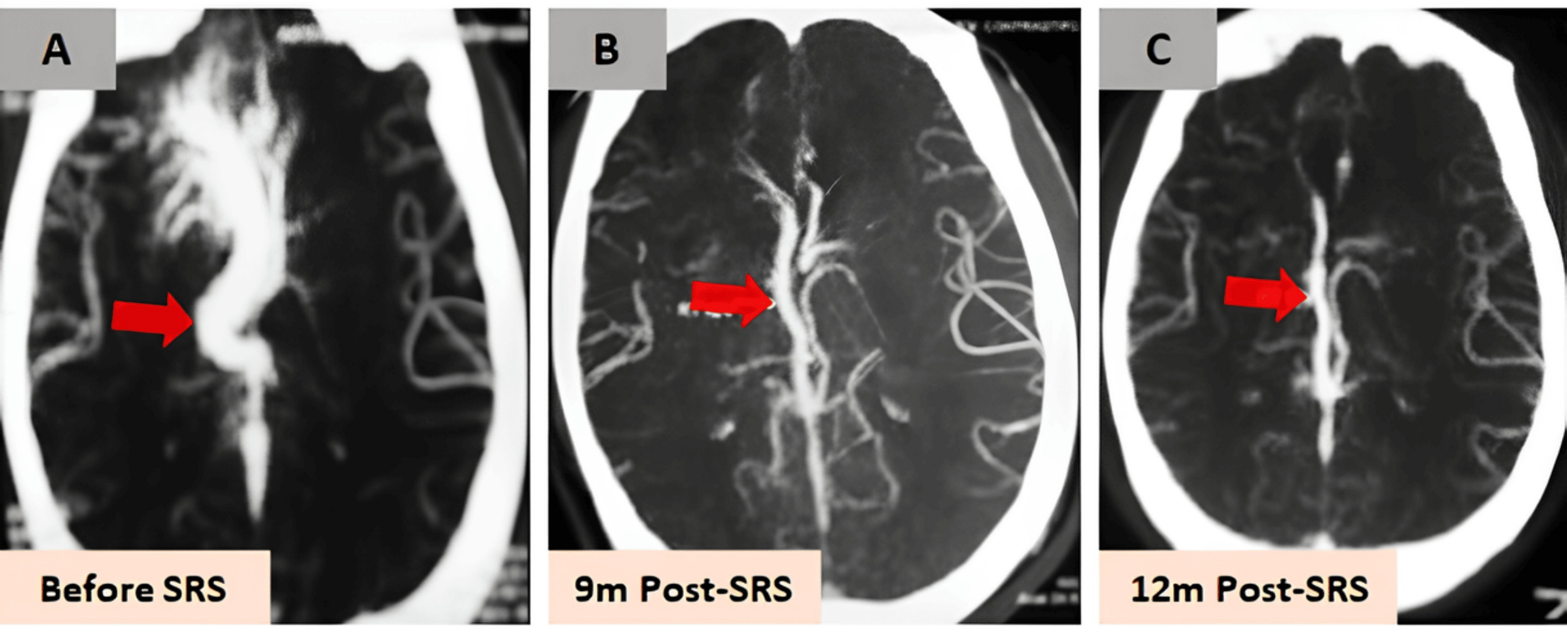
Progressive obliteration on CTA Serial CTA images before (A) and after SRS (B, C), illustrating progressive AVM nidus obliteration (red arrows). By nine months, CTA shows complete non-visualization of the arteriovenous malformation nidus, though mild dilation of the deep venous drainage system persists (red arrow). CTA, CT angiography; SRS, stereotactic radiosurgery

**Figure 4:**
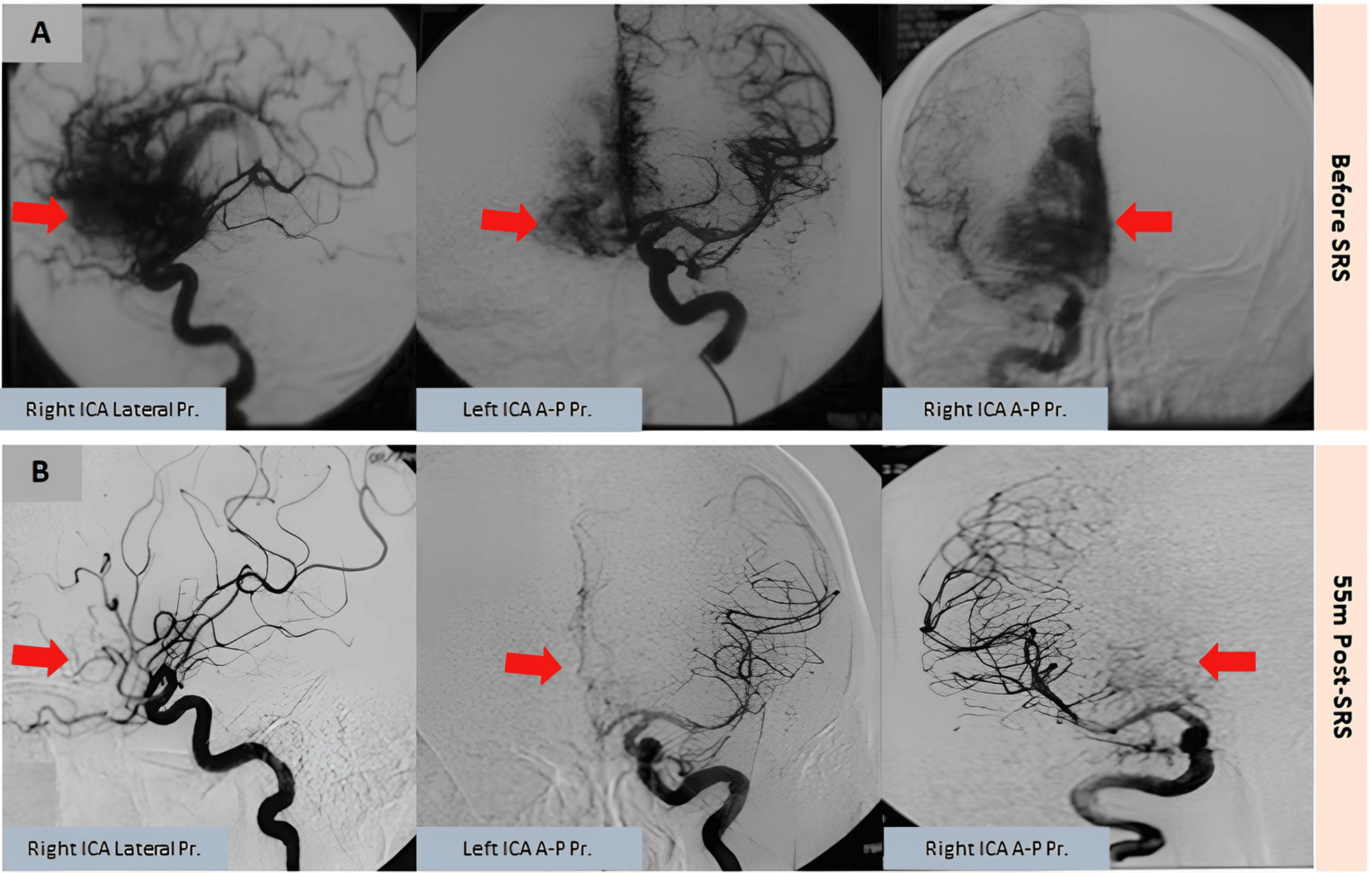
Complete AVM obliteration on DSA Pre- (A) and post-SRS (B) DSA confirming complete AVM obliteration (red arrows), with no residual nidus or abnormal shunting at 55 months post-SRS. AVM, arteriovenous malformation; DSA, digital subtraction angiography; ICA, internal carotid artery; SRS, stereotactic radiosurgery

Radiological follow-up showed a progressive response to treatment (Figures 2-4). By three months, CT imaging demonstrated a slight decrease in the size of the AVM nidus but significant vasogenic edema in the right frontal region. At seven months, MRI revealed a marked decrease in nidus size with persistent high T2 signal intensity, consistent with radiation-induced edema (not shown). By nine months, CTA showed complete non-visualization of the AVM nidus, although deep venous drainage remained mildly dilated.

By 12 months post-SRS, the patient was completely asymptomatic and neurologically intact. Serial imaging over the following months showed continued regression of the lesion. At 55 months, conventional angiography confirmed complete obliteration of the AVM, with no evidence of residual nidus or abnormal shunting. At his 64-month follow-up, he remained asymptomatic with a normal neurological examination, having experienced no further hemorrhagic events.

## Discussion

The case presented here underscores the diagnostic and therapeutic challenges associated with venous-predominant AVMs, particularly those that mimic DVAs. DVAs are typically considered benign vascular anomalies, but when an AVM presents with a venous-predominant nidus, the distinction becomes critical, as the management and prognosis differ significantly [3]. DVAs are characterized by a radial pattern of medullary veins converging into a single, larger draining vein, resembling a "caput medusae" (Medusa's head). DVAs are thought to represent a variation of normal venous drainage and are usually asymptomatic, requiring no treatment unless they are associated with other vascular anomalies or rare complications such as venous hypertension or thrombosis. In contrast, venous-predominant AVMs are a rare subtype of AVMs where the nidus (the central tangle of abnormal vessels) is predominantly composed of venous structures. Unlike DVAs, AVMs involve a direct connection between arteries and veins without an intervening capillary bed, leading to high-pressure blood flow and an increased risk of hemorrhage, seizures, or neurological deficits. The key difference lies in the hemodynamics and pathological nature of the lesions: DVAs are low-flow, low-pressure anomalies, while venous-predominant AVMs are high-flow, high-pressure lesions with a significant risk of rupture. The distinction between these two entities is critical because their management and prognosis differ significantly. DVAs are generally left untreated unless they cause symptoms or are associated with complications, as surgical or interventional treatment can disrupt normal venous drainage and lead to venous infarction. On the other hand, venous-predominant AVMs often require active intervention, such as endovascular embolization, surgical resection, or SRS, to reduce the risk of hemorrhage and other complications. Misdiagnosis can lead to either unnecessary intervention for a benign DVA or delayed treatment for a high-risk AVM, both of which can have serious consequences for the patient. In this case, the initial presentation with a thunderclap headache and subsequent imaging findings of SAH prompted further investigation, leading to the identification of a rare venous-predominant AVM.

The decision to proceed with SRS was based on the high surgical risk associated with the AVM's deep location and diffuse nature, as assessed by the Spetzler-Martin grading system [2] and the Lawton supplementary scale [4]. The Spetzler-Martin grade of 4/5 and the Lawton supplementary score of 2/5 indicated a high risk of neurological deficit with surgical intervention, making SRS a more favorable option. The Toronto scale further supported this decision, estimating a 31.6% probability of neurological deficit with surgery [1,6,7].

SRS, utilizing methods such as proton beam, gamma knife, linear accelerator (LINAC), or CyberKnife, is generally used for small, well-defined lesions [7]. These smaller lesions typically do not contain normal brain tissue that could be adversely affected by radiation, potentially leading to the formation of epileptogenic gliotic tissue. This approach is currently the favored treatment for most patients, unless specific clinical reasons favor surgical intervention, such as addressing an AVM during hematoma removal or patient preference after thorough consultation. Following SRS, which can take up to four years to fully eliminate the nidus, there is an annual hemorrhage risk estimated at 4.8%-7.9% for the first two years, decreasing to 2.2%-5% in the subsequent three years [5,8]. Complete obliteration of the nidus nearly eliminates the risk of future hemorrhages, although Maruyama et al. noted that six out of 250 patients experienced bleeding after their AVMs were considered cured [9]. For small, compact nidi in non-critical brain areas, complete obliteration is achievable in over 90% of cases, though success rates drop for larger AVMs, which often necessitate lower radiation doses to prevent radionecrosis-related bleeding. The therapeutic effect of radiosurgery on AVMs is believed to result from radiation-induced damage to endothelial cells, causing vascular smooth muscle overgrowth and subsequent vessel blockage. Post-treatment monitoring typically includes annual MRI scans to assess nidus obliteration, a process that usually takes two to three years but can extend to four years. According to a meta-analysis by van Beijnum et al., around 40% of AVMs treated with radiosurgery achieve complete obliteration within roughly 24 months [10]. If MRI scans no longer show flow voids in the treated area, a DSA is recommended to confirm obliteration. If obliteration is not achieved within four years, retreatment with radiosurgery may be considered.

In this case, the patient underwent linac-based SRS with a marginal dose of 25.0 Gy, which resulted in complete obliteration of the AVM nidus by 55 months post-treatment, as confirmed by conventional angiography. This outcome is consistent with previous studies reporting long-term obliteration rates of 70%-80% for AVMs treated with SRS [11].

The patient's clinical course was complicated by radiation-induced vasogenic edema, a known side effect of SRS, which manifested as intermittent headaches and vomiting. These symptoms were managed successfully with steroids and diuretics, and the patient remained neurologically intact throughout the follow-up period. The resolution of symptoms and the absence of further hemorrhagic events at 64 months post-SRS highlight the long-term efficacy and safety of this treatment modality.

This case also emphasizes the importance of serial imaging in monitoring the response to SRS. Progressive reduction in the size of the AVM nidus was observed on CT and MRI, with complete non-visualization of the nidus on CTA by nine months post-SRS. The persistence of mild dilation in the deep venous drainage system, however, suggests that while the AVM nidus was obliterated, some residual venous changes may persist, which is consistent with findings from other studies [7,11,12].

## Conclusions

In conclusion, this case illustrates the successful management of a rare venous-predominant AVM mimicking a DVA using SRS. The long-term follow-up confirms the efficacy of SRS in achieving complete obliteration of the AVM while minimizing the risk of neurological deficits. This case also highlights the importance of a multidisciplinary approach in managing complex AVMs, particularly those in high-risk locations.
